# Determining factors of sports dropout of young scholars: a cross-sectional analysis in the 8–13 year age group

**DOI:** 10.3389/fspor.2024.1330346

**Published:** 2024-07-23

**Authors:** Andrea Pisaniello, Alessandro Figus, Simone Digennaro, Diana Spulber

**Affiliations:** ^1^ Department of Human, Social and Health Sciences, University of Cassino and Southern Lazio, Cassino, Italy; ^2^ Department of Education Sciences, University of Genoa, Genoa, Italy

**Keywords:** pedagogy, sport, inclusion, dropout, early adolescence

## Abstract

This article investigates the phenomenon of sports abandonment among young scholars aged between 8 and 13 years. Regardless of the growing awareness of the importance of sport and physical activity during childhood and adolescence, this theme must be adequately explored in the scientific literature. Our study addresses this gap through a cross-sectional research design, tracking and analyzing data from a cohort of young athletes over one year. The main objective of our study is to identify the determinants leading to sports dropout in this specific age group. We looked at several possible causes through a multivariate analysis, including social pressures, parental expectations, time conflicts, physical and psychological stress, and lack of enjoyment. The results show a significant attrition rate, with psychosocial factors emerging as the most influential in determining whether a young person will continue or stop their participation in sport. Furthermore, our study highlights the importance of targeted interventions and preventive strategies that promote a positive, inclusive, and balanced sports environment for adolescents. These interventions can be particularly effective when implemented by coaches, parents and others involved in youth sports education. Finally, this paper discusses the implications of the findings for sports professionals, physical educators, and public policy makers. It highlights the need for more effective support policies and innovative pedagogical approaches to promote sporting persistence during adolescence. Our findings can serve as a starting point for further research in this field, helping to build a future where young people can enjoy the many benefits of sport and physical activity.

## 1 Introduction

Physical activity and sports participation have a profound impact on the health and development of young people. The positive effects of physical activity in the pediatric population have been widely documented, including improvements in cardiovascular health, bone health, body composition, and mental health (1). Beyond the physical benefits, sports participation can also offer several psychosocial benefits, including improving self-esteem, developing problem-solving skills, and building social bonds (2, 3).

Addressing the rate of sports dropout among young people, particularly during adolescence, is a pressing concern that warrants attention. Fraser-Thomas et al. (4) shed light on the prevalence of sports dropout among young athletes, highlighting its significance. Additionally, Pisaniello, Figus, and Digennaro (5) underscore the severity of this issue, stating, “This growth is not so clear when we go back to adolescence and youth, where unfortunately the highest dropout rates are recorded. The age between 10 and 16 is the critical period in which abandonment occurs. Furthermore, in the phase of compulsory secondary education, a percentage is established which always remains below the threshold of 50%” (5). Their research delves into the statistical data on youth dropout rates within the European context, providing valuable insights into the dynamics of sports dropout among adolescents (5) and youth in Europe. Understanding these regional statistics is crucial for devising effective interventions and strategies tailored to the unique challenges faced by young athletes in European societies.

One of the least studied aspects of sports dropout concerns preadolescents and adolescents between the ages of 8 and 13, representing a crucial transition phase from early childhood to adolescence. Often overlooked in the literature in favour of older adolescent groups, this age group presents unique challenges and opportunities regarding sports involvement. While individuals in this age group are less prone to performance pressures than their older peers, they may be more susceptible to external influences such as parental expectations or peer pressure (6).

The importance of keeping young people in sports during early adolescence cannot be emphasised enough. In this time of rapid cognitive, physical, and emotional development, sports experiences can provide young people with a structured environment to face and overcome challenges, learn the value of teamwork, and develop resilience (7). However, young people disengage from the sport at this crucial stage. In that case, they miss out on the opportunity to benefit from these positive experiences, which could impact their overall health and long-term well-being.

Sporting activities are voluntary, and the enjoyment and emotional appeal, and relative accessibility of sports make them an appealing force; the loss of enjoyment and the lack of interest can be a cause of sports dropout. Various research has attempted to identify the reasons for sports abandonment, highlighting factors ranging from lack of enjoyment to performance pressure to logistical challenges such as time and costs (8–11).

However, few studies have explicitly focused on the 8–13 age group despite this representing a critical period in which young people are particularly vulnerable to sports disaffection (12, 13).

There is little research about the correlation between cost reasons and sports dropout. In their longitudinal research about hockey dropouts made in 2017 and 2018, Owen found that the reasons for dropout were medical/age, change in circumstances, high cost, and lack of time (14).

In the context of Italy, factors leading to sports dropout among children and adolescents aged 8–13 include academic pressure, economic costs, lack of family support, competing interests, negative experiences such as bullying, health issues, lack of enjoyment, family relocations, inadequate facilities, and peer influence. These factors, individually or collectively, can significantly impact the continuation of sports participation among young individuals. Through cross-sectional analysis, we aim to identify this group's main causes of abandonment and to provide ideas for targeted interventions and preventive strategies.

By incorporating these methods and based on solid methodological references, this study aims to provide a detailed and sustained insight into the reasons for sports dropout in the specific age group.

The environment in which the sport is practised also plays a crucial role. Coaches, parents, and peers can significantly influence a youth's decision to continue or leave sport (6). While a supportive and motivating coach can encourage persistence, unrealistic expectations or a hostile climate can turn young people away from the sporting environment.

Socialisation is an important factor at this age; in fact, sport could serve as a springboard for making new friends and expanding social networks, in the meantime the absence of involvement of peers of parents can positively impact on sport dropout (9, 15).

The cultural and social context adolescents live in can influence their sports experiences. For example, gender-related cultural expectations can influence boys' and girls' sporting opportunities and participation, while socioeconomic factors can limit access to sporting facilities and activities.

Given the importance of understanding and addressing the causes of sports dropout among children and adolescents aged 8–13, this study aims to fill existing gaps in the literature. Through multifactorial approach, we will seek to offer a detailed insight into the factors that lead to sports dropout to provide practical insights for coaches, educators, and sports policymakers.

## 2 Materials and methods

### 2.1 Participants

We designed our sample of research in two steps. As a first step, we used the snowing ball guide given the participants’ peculiarities, especially their age and occupation (16). While some researchers support the definition of the snowball method as a sampling method in which a respondent provides the researcher with the name of at least one other potential respondent (17, 18), in our research we started with the national Italian Sports Center (CSI) which in turn contacted the regional CSI which contacted the gyms.

To reach a larger number of participants, the study involved a collaboration between gyms, schools, and a multicenter approach across the national territory. Utilizing a combined methodological approach, a network was established involving three universities and provincial committees of the CSI. Participants were recruited freely with parental consent, ensuring a diverse and representative sample for the administration of the questionnaire. This extensive network facilitated access to a broad demographic of children and adolescents, enhancing the study's reliability and comprehensiveness.

The sample consisted of 6,783 Italian students, selected to represent a diverse range of gender, socioeconomic, and geographic backgrounds. This selection was based on the sampling techniques guidelines outlined by Dillman et al. (19), specifically the second step. The participants were young students aged 8–13 years.

### 2.2 Procedure

Interviewers previously trained in an earlier study and questionnaire administrators trained during a dedicated meeting were involved to ensure data collection accuracy and adherence to procedures. This prior training equipped the interviewers with the necessary skills and knowledge, ensuring that the data collection process was consistent and reliable across all locations. The comprehensive training session covered all aspects of the questionnaire administration, thereby standardizing the approach and minimizing any potential biases or errors in data gathering.

These students were administered a structured questionnaire, which included multiple-choice questions and questions based on the Likert scale, a method recognised for reliability in measuring perceptions and opinions (20).

The questionnaire was structured into three distinct sections, each with a specific objective; this will permit us to understand the dynamics underlying the phenomenon of sports dropout in Italy.

The data collection was conducted by adopting a complex research strategy involving an online questionnaire distributed via the Qualtrics platform and a more traditional data collection based on the administration of paper questionnaires at educational institutions and Sports Associations.

Participants received only preliminary information relating to the research objectives and no indication of merit. This choice was made to ensure the integrity and objectivity of the responses provided by the participants.

Data collection was carried out following strict anonymity and confidentiality policies. The data collected will be used exclusively for research and non-commercial purposes, in aggregate and anonymous form, in full compliance with the Code regarding the protection of personal data (Legislative Decree 196/2003), updated with the new legislative decree (Legislative Decree. Lgs. 101/2018) to adapt Italian legislation to the European privacy regulation (EU Reg. no. 679/2016, GDPR).

• Personal Data Section: This section collected demographic information on the respondents, offering a cross-section of the sample population. This data is essential to contextualize subsequent responses and better understand demographic variables that may influence sport dropout. Demographic variables included, for example, age, gender, place of residence and level of education.
• Sports Activity Section: The core of the questionnaire. Here, participants were questioned about their past and present sporting experiences. Detailed questions explored the type of sport practised, the duration of the activity, the weekly frequency, the reasons for any abandonment and any perceived barriers to practice. This section has provided fundamental data to outline the main causes of sports abandonment and identify any areas for intervention.
• Section Information on the Family and Mode of Sports Practice: This section aimed to investigate the influence of the family environment and the methods of sports practice on the dropout phenomenon. Questions were asked regarding perceived family support, the presence of other family members active in sport, and how sport had been practiced (e.g., independently, in a club, in a federation). Understanding the influence of family context and practice patterns helps define the extent to which these factors may act as facilitators or barriers to sporting continuity.

### 2.3 Data analysis

The collected data were organized in a spreadsheet and meticulously double-checked for typos and errors. To ensure a thorough analysis, the data were stratified by both gender and age. Dropout rates were further examined across these demographic segments.

The quantitative data were subjected to statistical analysis, following the guidelines proposed by Field (21) to identify trends in the analyzed sample. Regarding the qualitative data, a thematic analysis was adopted following the method proposed by Braun & Clarke (22), which allows us to identify, analyze and report patterns or themes in the data.

## 3 Results

The data analysis showed a prevalence of respondents in the 13-year age group, constituting almost a third (29%) of the total sample. This was closely followed by 12-year-old adolescents, who accounted for just over a fifth (23%). Eleven-year-olds comprised 18%, while the youngest age groups (8 and 9 years old) represented 9% and 10%, respectively. This distribution indicates greater participation in the questionnaire by older adolescents than younger ones (see Table 1).

**Table 1:** Distribution of the respondents (3,449 boys, 3,325 girls) according to age and sequentially divided for gender.

Distribution of respondents			
Age	*n*	Boys	Girls
8	595 (9%)	327 (55%)	268 (45%)
9	663 (10%)	351 (53%)	312 (47%)
10	782 (12%)	430 (55%)	352 (45%)
11	1,223 (18%)	624 (51%)	599 (49%)
12	1,534 (23%)	767 (50%)	767 (50%)
13	1,986 (29%)	953 (48%)	1,033 (52%)

The sample was almost equally divided between genders, slightly biased towards men. Males accounted for 51%, while females were slightly fewer, accounting for 49%. A small segment (0.13%) had not specified gender (Table 1).

The analysis unveiled that the primary class (1st grade) exhibited the highest prevalence, accounting for 23% of the total. Subsequently, the intermediate grades, 2nd and 3rd, demonstrated approximate representation levels at 22% and 21%, respectively. In contrast, the lower grade categories exhibited lower proportions, with the 2nd grade at 3% and the 3rd, 4th, and 5th grades at 10%, 11%, and 10%, respectively.

A consistent majority of 57% dedicates between 1 and 2 h a day to studying in the afternoon. However, a small but significant 3% dedicate more than 5 h daily to studying.

When we look at how students get to school, the car emerges as the predominant means of transport, with 49%. This may reflect a combination of socio-economic and practical factors. In contrast, a notable 29% walk to school highlights the possible proximity of school homes and, perhaps, an urban context. Public transport, including buses and metro, is used by 15% of students.

Regarding sports, most students, 74%, are actively involved in sports activities. However, there is 11% who currently do not engage in any sports practice and another 14% who stated that they have stopped practicing sports in the past.

In summary, while a large percentage of students actively engage in study and sport and have specific modes of transportation to get to school, significant segments choose or are forced to do otherwise.

Looking closely at students' daily study habits, an interesting scenario emerges. A significant percentage, 36%, declare that they study only between 0 and 1 h a day. A further 31% of students say they study approximately 2 h daily, which could be considered an average daily study duration. Moving on to a greater commitment, 21% of students dedicate 3 h daily to studies. Only 11% of students study for 4 h or more daily. Finally, a small percentage, 2%, did not specify the study hours.

We can see several recurring themes as we explore why people play sports. As many as 61% of the majority see practising sports as maintaining physical well-being. Aesthetic beauty is another important reason, with 44% practicing sports to improve their physical appearance.

**Figure 1:**
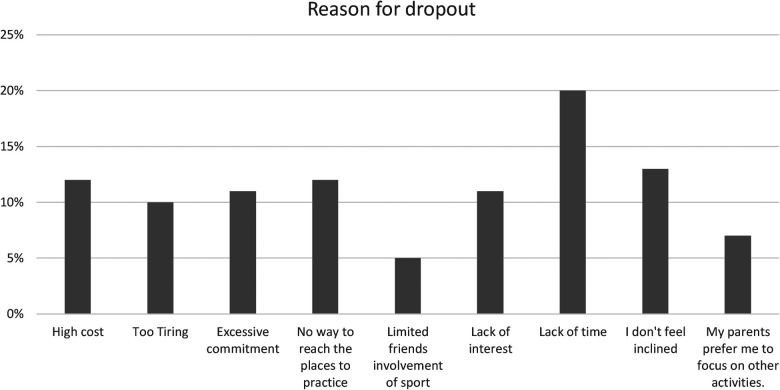
Principal reasons for sports dropout among respondents.

**Figure 2:**
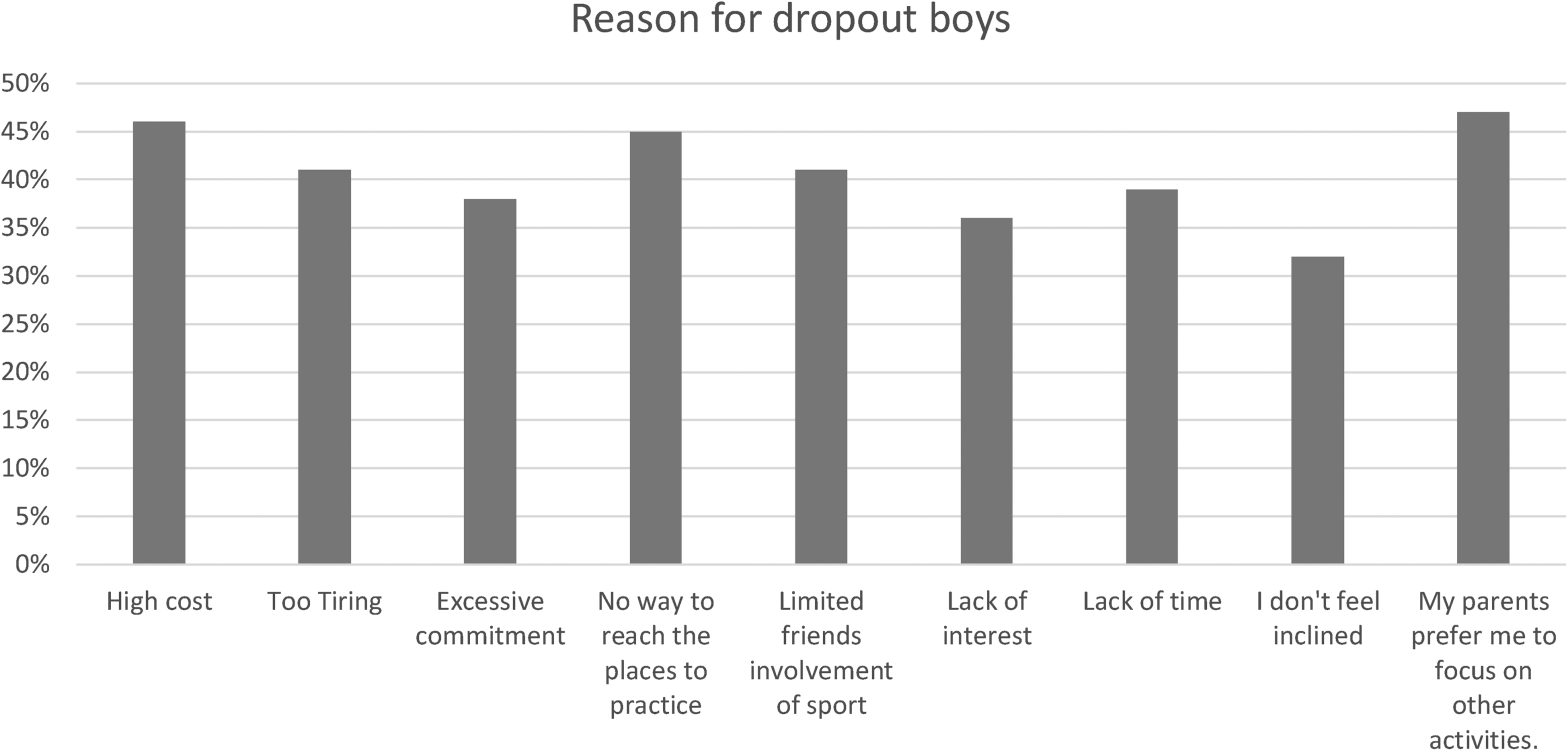
Principal reasons for sports dropout among boys.

**Figure 3:**
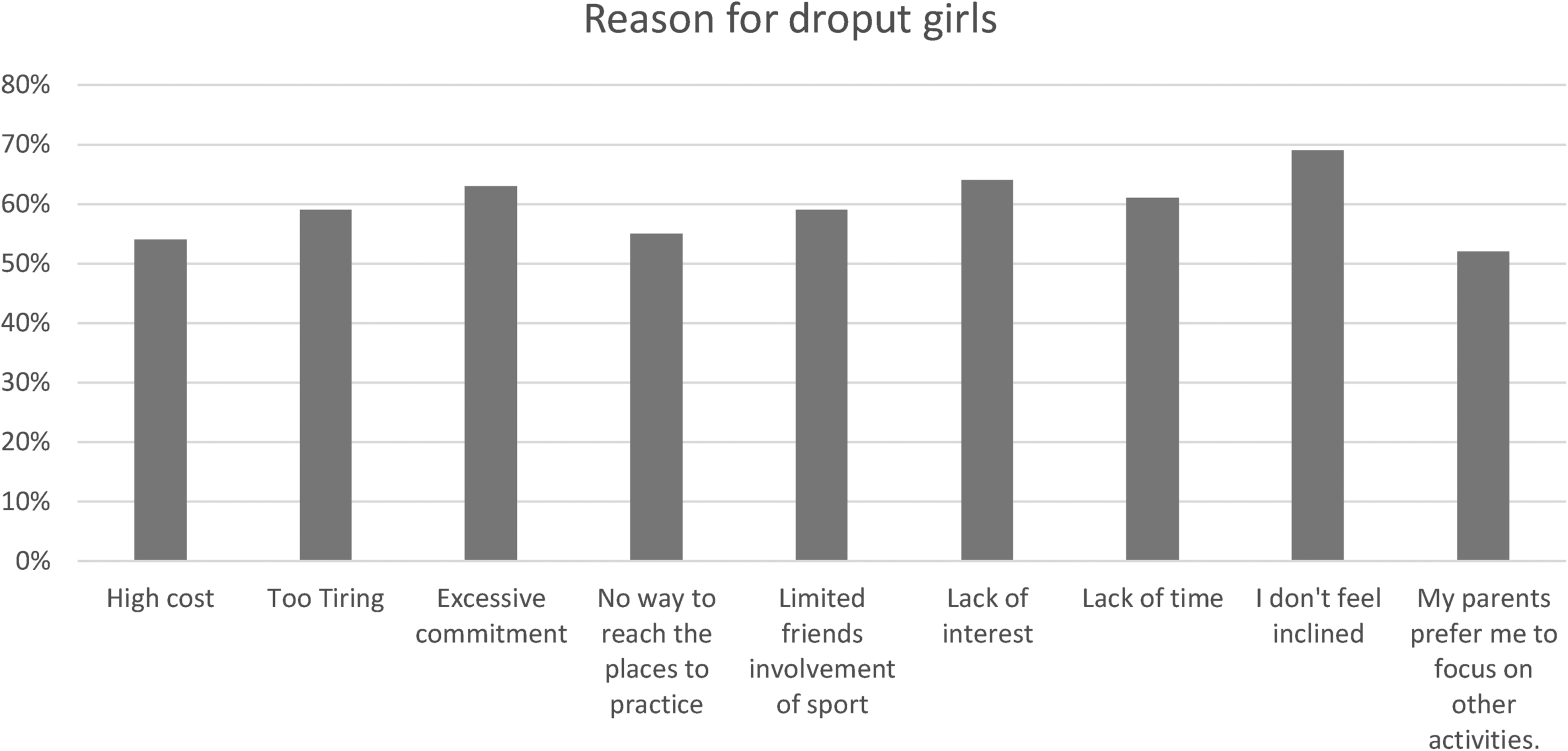
Principal reasons for sports dropout among girls.

Furthermore, 88% participate in sports simply because they derive pleasure and passion. The sociocultural aspect of sport cannot be overlooked, given that 58% of individuals are motivated by socialization. Personal aspirations motivate 52%, and a high percentage, 91%, feel a sense of obligation towards practising sport, which could arise from societal or personal pressures. From the results it is evident that 20% (see the Figure 1) of the respondents drop out of sports due to a lack of time. Of this 20%, 61% are girls (See the Figure 3) and 31% are boys (See the Figure 2).

The phenomenon of dropping out from sports activities is influenced by high cost 12% (see the Figure 1) of which 54% are girls (see the Figure 3) and 46% are boys 46% (see the Figure 2).

When examining the duration of practice of the main sport, 23% of people have practised their main sport for 1–2 years, while 21% have practised for 2–4 years. Interestingly, 20% of people have less than one year of experience in sports. A percentage of 16% have been practising for 4–6 years, and 13% have practised their main sport for 6–8 years.

## 4 Discussion

One of the least studied aspects of sports dropout concerns preadolescents and adolescents between the ages of 8 and 13, representing a crucial transition phase from early childhood to adolescence. Often overlooked in the literature in favour of older adolescent groups, this age group presents unique challenges and opportunities regarding sports involvement. While individuals in this age group are less prone to performance pressures than their older peers, they may be more susceptible to external influences such as parental expectations or peer pressure (6).

The evidence emerging from our study reveals a contrasting landscape regarding perceptions of sport and community participation. Although the positive evaluation of sport and its relevance for individual and collective well-being is universally recognized, barriers still hinder active and continuous participation.

First, the importance of sport for physical and mental health was highlighted by our data, in line with previous studies (23). Physical exercise is important not only for offering protection from a range of chronic diseases but is crucial in promoting mental health and reducing the risk of conditions such as depression (24).

However, despite this widespread awareness, effective participation still needs to be solved. The barriers identified in our study, such as lack of facilities, associated costs, and lack of time or motivation, align with previous literature (25). While varying by socioeconomic and geographic conditions, these obstacles represent a persistent challenge in many communities.

The systematic research literature confirms our results about the reasons for dropout like High 11% cost and lack of time or incompatible schedule 27%, lack of Interest (9, 26). The logistic accessibility is another important indicator that can impact positively sport dropout in our research the Item Difficulty reaching spaces is 23%t and is in line with the research of Sevilmis and others about gym renewing membership for fitness clubs were the criteria with the highest weight. In our research, 20% of participants indicated the cause of dropout the lack of interest which is in line with the research of Van Yperen about predictors of dropout from organized football in which the lack of enjoyment was a major predictor of both short-term (6 months) and long-term (4 years) dropout.

A further reflection emerges on the need to make physical activity a more inclusive and community experience. Creating sports groups and organising events at a local level could not only break down some of the perceived barriers but also strengthen social bonds, which are essential for a resilient and cohesive community.

Our study highlights the importance of a holistic approach to promote active sports participation. While awareness of the benefits of sports is high, addressing real and perceived barriers through targeted interventions is crucial. This will require a collective effort from public bodies, sports associations, and the community to ensure that physical activity becomes accessible and shared by all.

As we have seen, the data from our study raises an alarm on sports dropout among young people that cannot be ignored.

But as often happens regarding social dynamics, the situation is not uniform throughout the territory. Analyzing the data at a regional level, a notable disparity emerges between Northern and Southern Italy. While the North has slightly lower dropout rates, the South shows figures that raise further concerns. These differences can be attributed to a combination of factors, from the availability of sports infrastructure to local sports culture and public investment in this sector.

Considering this information, the solution cannot be univocal or generic. Italy needs a widespread, targeted intervention based on concrete data. The priorities emerge clearly:

First is the infrastructure. Active sporting participation cannot be hoped for without adequate facilities. The strengthening and modernization of sports infrastructures, especially in the most lacking regions, become essential.
Second, education. Not only training for coaches or physical education teachers but also a renewed commitment to integrating sports culture in schools, showing young people the importance and benefits of regular sports practice.

Finally, community and family support. A family that supports sports practice can make the difference in a young person's choice to continue or abandon sports. Likewise, active and engaged communities can create a stimulating and inclusive environment.

In conclusion, while the current data on sports abandonment in Italy may seem discouraging, it also offers the opportunity to reflect, plan and act with the right strategies and an approach based on research and collaboration.

Recommendations:

I. Improve facilities: Investing in high-quality facilities and ancillary services could encourage participation.
II. Interventions at the school level: Promoting more sporting activities in schools could instil the importance of sports in younger generations.
III. Subsidies and financial aid: Offering discounts, scholarships or other subsidies could play sports more accessible to a wider audience.
IV. Encourage group practice: Creating sports groups or community events could increase participation and strengthen a sense of community.
V. Integration of technology: Developing apps or other technological solutions can modernize and personalize the approach to sport, making it more attractive to new generations.
VI. Flexibility: Considering extended or variable hours for athletic facilities can help accommodate the varying needs of individuals.

While many people enjoy and want to participate in sports, some barriers and challenges can hinder them. Data analysis suggests many opportunities exist to promote and incentivize broader and more sustainable sports participation with the right initiatives and investments.

## 5 Conclusions

The depth of our investigation into sports perception and participation offered a clear and detailed overview of the current situation in each community. A framework of universal recognition of the benefits of sport for physical and psychological well-being has emerged but is counteracted by various obstacles that limit access and regular practice.

Although awareness is high regarding the positive impacts of physical activity on health, logistical barriers such as lack of adequate facilities, associated costs and limited time remain dominant concerns for many. Psychological barriers, such as lack of motivation or perceived competence, are equally relevant. It is interesting to note how, in a society where technology permeates every aspect of daily life, digital solutions are seen as potential aids but not as substitutes for real, physical engagement.

Based on the analyses presented, we can draw the following conclusion:

Practicing sports is seen extremely positively by most people who participated in the research, who recognize numerous physical and mental health benefits. In fact, according to Warburton, Nicol, and Bredin (23), regular physical activity can “prevent chronic diseases, increase longevity, improve quality of life, reduce the risk of injury and promote mental well-being”. However, despite widespread awareness of the benefits, significant barriers prevent many individuals from participating in sport regularly.

The main concerns include the lack of accessible facilities, associated costs and lack of time or motivation. These barriers align with a study by Trost et al. (25) who identified similar barriers, particularly lack of access and financial limitations, as main reasons for non-participation in physical activity.

Policymakers and sports practitioners should focus on reducing these barriers to maximize sport participation and take advantage of its public health benefits. This could include investments in local sports infrastructure, subsidy programs to reduce the costs associated with playing sports, and awareness campaigns.

In conclusion, although interest in sport and awareness of its benefits are high, a collective commitment from public, private and community bodies is essential to ensure these positive attitudes translate into effective and widespread sporting practice.

To effectively address these challenges, a multidimensional approach is required. Promoting awareness, while essential, is not sufficient. It is essential to ensure accessibility to facilities, break down economic barriers and create opportunities for training and sports education, creating a more inclusive and integrated sports culture.

In summary, sport is a powerful tool for individual well-being and social cohesion. As such, efforts to broaden its reach and penetration into society should be a priority. The key is the combination of awareness, accessibility and interventions tailored to the specific needs of different communities.

## Data Availability

The raw data supporting the conclusions of this article will be made available by the authors, without undue reservation.

## References

[1] Janssen I LeBlanc AG. Systematic review of the health benefits of physical activity and fitness in school-aged children and youth. Int J Behav Nutr Phys Act. (2010) 7(1):40. 10.1186/1479-5868-7-40 20459784 PMC2885312

[2] Eime RM Young JA Harvey JT Charity MJ Payne WR. A systematic review of the psychological and social benefits of participation in sport for adults: informing development of a conceptual model of health through sport. Int J Behav Nutr Phys Act. (2013) 10(1):135. 10.1186/1479-5868-10-135 24313992 PMC4028858

[3] Eime RM Young JA Harvey JT Charity MJ Payne WR. A systematic review of the psychological and social benefits of participation in sport for children and adolescents: informing development of a conceptual model of health through sport. Int J Behav Nutr Phys Act. (2013) 10(1):98. 10.1186/1479-5868-10-98 23945179 PMC3751802

[4] Fraser-Thomas J Côté J Deakin J. Understanding dropout and prolonged engagement in adolescent competitive sport. Psychol Sport Exerc. (2008) 9(5):645–62. 10.1016/j.psychsport.2007.08.003

[5] Pisaniello A. Figus A Digennaro S. Determinants and reasons for participation and drop out in sports activities in Italy. Italian Journal of Health Education, Sports and Inclusive. (2023) 7(1):1–2. 10.32043/gsd.v7i1.843

[6] Gould D. Early sport specialization: a psychological perspective. J Phys Educ Recreat Dance. (2010) 81(8):33–7. 10.1080/07303084.2010.10598525

[7] Holt NL Neely KC Slater LG Camiré M Côté J Fraser-Thomas J A grounded theory of positive youth development through sport based on results from a qualitative meta-study. Int Rev Sport Exerc Psychol. (2017) 10(1):1–49. 10.1080/1750984X.2016.1180704 27695511 PMC5020349

[8] Côté J Lidor R Hackfort D. ISSP position stand: to sample or to specialize? Seven postulates about youth sport activities that lead to continued participation and elite performance. Int J Sport Exerc Psychol. (2009) 9(1):7–17. 10.1080/1612197X.2009.9671889

[9] Monteiro D Determinants and reasons for dropout in swimming—systematic review. Sports. (2017) 5(3):50. 10.3390/sports5030050 29910410 PMC5968952

[10] Van Yperen NW Jonker L Verbeek J. Predicting dropout from organized football: a prospective 4-year study among adolescent and young adult football players. Front Sports Act Living. (2022) 3:1–7. 10.3389/fspor.2021.752884 35112082 PMC8801566

[11] Marinho D Moutao J Vitorino A Antunes R Cid L. Reasons for dropout in swimmers, differences between gender and age and intentions to return to competition. J Sports Med Phys Fitness. (2018) 58(1–2):180–92. 10.23736/S0022-4707.17.06867-0 28474870

[12] Wagnsson S Gustafsson H Libäck J William Podlog L. Lessons learned from a multi-level intervention program to reduce Swedish female floorballers’ dropout rate. J Sport Psycho Action. (2021) 12:226–44. 10.1080/21520704.2020.1850576

[13] Back J Johnson U Svedberg P McCall A Ivarsson A. Drop-out from team sport among adolescents: a systematic review and meta-analysis of prospective studies. Psychol Sport Exerc. (2022):102205. 10.1016/j.psychsport.2022.102205

[14] Owen KB Foley BC Eime R Rose C Reece LJ. Participation and dropout of hockey New South Wales participants in 2017 and 2018: a longitudinal study. BMC Sports Sci Med Rehabil. (2022) 14(103):1–8. 10.1186/s13102-022-00494-2 35676741 PMC9174916

[15] Jaf D Wagnsson S Skoog T Glatz T Özdemir M. The interplay between parental behaviors and adolescents’ sports-related values in understanding adolescents’ dropout of organized sports activities. Psychol Sport Exerc. (2023) 68:1–10. 10.1016/j.psychsport.2023.102448 37665898

[16] Parker C Scott S Geddes A. Snowball sampling. In: Atkinson P Delamont S Cernat A Sakshaug J.W Williams RA, editors. SAGE Research Methods Foundations. London: SAGE Publications (2019). p. 1–13. 10.4135/978152642103683171

[17] Cohen N Arieli T. Field research in conflict environments: methodological challenges and snowball sampling. J Peace Res. (2011) 48(4):423–35. 10.1177/0022343311405698

[18] Bhattacherjee A. Social Science Research: Principles, Methods, and Practices. Tampa, FL: Creative Commons Attribution-NonCommercial-ShareAlike (2012).

[19] Dillman DA Smyth JD Christian LM. Internet, Phone, Mail, and Mixed-Mode Surveys: The Tailored Design Method. Hoboken, NJ: Wiley (2014).

[20] DeVellis RF. Scale Development: Theory and Applications. New York: SAGE Publications, Inc. (2016).

[21] Field A. Discovering Statistics Using IBM SPSS Statistics. Thousand Oaks: SAGE Publications (2013).

[22] Braun V Clarke V. Using thematic analysis in psychology. Qual Res Psychol. (2006) 3(2):77–101. 10.1191/1478088706qp063oa

[23] Warburton DER Nicol CW Bredin SSD. Health benefits of physical activity: the evidence. CMAJ. (2006) 174(6):801–9. 10.1503/cmaj.051351 16534088 PMC1402378

[24] Rebar AL Stanton R Geard D Short C Duncan MJ Vandelanotte C. A meta-meta-analysis of the effect of physical activity on depression and anxiety in non-clinical adult populations. Health Psychol Rev. (2015) 9(3):366–78. 10.1080/17437199.2015.1022901 25739893

[25] Trost SG Owen N Bauman AE Sallis JF Brown W. Correlates of adults’ participation in physical activity: review and update. Med Sci Sports Exerc. (2002) 34(12):1996–2001. 10.1097/00005768-200212000-00020 12471307

[26] Crane J Temple V. A systematic review of dropout from organized sport among children and youth. Eur Phy Educ Rev. (2015) 21:114–31. 10.1177/1356336X14555294

